# Supplementary material for: Acceptability and feasibility of an acceptance and commitment therapy-based guided self-help intervention for weight loss maintenance in adults who have previously completed a behavioural weight loss programme: the SWiM feasibility study protocol
Source: BMJ Open. 2022 Apr 18;12(4):e058103. doi: 10.1136/bmjopen-2021-058103 (PMC9020279; doi:10.1136/bmjopen-2021-058103)
Supplement: Supplementary data [file bmjopen-2021-058103supp001.pdf]

MRC Epidemiology Unit | UNIVERSITY OF CAMBRIDGE | SWiM SUPPORTING WEIGHT MANAGEMENT

Supporting Weight Management (SWiM) Feasibility Study

Participant study number:

CONSENT FORM

Version 0.7 (15/04/2021)

REC Ref:21/EE/0024 IRAS ID: 279784 Chief Investigator: Dr Amy Ahern

Please initial each box

1. I confirm that I have read and understood the information sheet **version 0.9, 15/04/2021** for the above study and have had the opportunity to consider the information, ask questions and have had these answered satisfactorily. ☐
2. I understand that my participation is voluntary and that I am free to withdraw at any time, without giving any reason and without my medical care being negatively affected or my legal rights. ☐
3. I understand that the information collected about me can be stored by the MRC Epidemiology Unit, University of Cambridge. ☐
4. To access the SWiM intervention, I give permission for my name and email address to be provided to Cauldron, the website developer, to create an account for me to access the web-based programme. ☐
5. I understand that I will not benefit financially if this research leads to the development of a new treatment or test. ☐
6. I agree to take part in the above study ☐

OPTIONAL:

	Yes	No
11. I agree to take part in focus groups and for such discussions to be recorded.	☐	☐
12. I agree to take part in a semi-structured interview	☐	☐
13. I understand that my pseudonymised data will be used to support other research in the future, and may be shared anonymously with other researchers. This could include collaborators overseas or in the commercial sector.	☐	☐
14. I am willing to be contacted again in the future about further follow up for this study or any future research.	☐	☐

White copy for the researcher, yellow for the GP, pink for the participant

--	□□ □□ □□□□	----------------------
Full name of Participant (BLOCK CAPITALS)	Date (DD MM YYYY)	Signature
--	□□ □□ □□□□	----------------------
Full name of Researcher	Date (DD MM YYYY)	Signature

Ahern AL, *et al. BMJ Open* 2022; 12:e058103. doi: 10.1136/bmjopen-2021-058103
