# Supplementary material for: Acceptability and feasibility of an acceptance and commitment therapy-based guided self-help intervention for weight loss maintenance in adults who have previously completed a behavioural weight loss programme: the SWiM feasibility study protocol
Source: BMJ Open. 2022 Apr 18;12(4):e058103. doi: 10.1136/bmjopen-2021-058103 (PMC9020279; doi:10.1136/bmjopen-2021-058103)
Supplement: Supplementary data [file bmjopen-2021-058103supp002.pdf]

SWIM

SUPPORTING WEIGHT MANAGEMENT

My Weight Maintenance Plan

Losing weight is hard and you should be proud of what you have achieved during your programme.

Preventing weight regain is also hard because our biology and our environment push us to gain weight.

One of the key things that successful weight maintainers do is make a plan for how they will continue to manage their weight.

To make a weight maintenance plan, it is helpful to think about what you did to lose weight.

Maybe you reduced snacking between meals

Maybe you went for a walk every day

List the effective changes you made below:

During my weight loss programme I made the following changes:

➔

➔

➔

➔

Look at your list of things that helped you to lose weight.
What will you do to help stay on track?

To help me stay on track I will:

➔

➔

➔

➔

It is a good idea to continue to monitor your weight and to think about what you will do if your weight creeps up again.

If I notice changes in my weight, I will:

➔

➔

➔

➔
